# IMAD: flexible annotation of microarray sequences

**DOI:** 10.1186/1753-6561-3-S4-S2

**Published:** 2009-07-16

**Authors:** Dennis Prickett, Michael Watson

**Affiliations:** 1 Bioinformatics Group, Institute for Animal Health (IAH), Compton, Newbury, RG20 7NN, UK

## Abstract

**Background:**

Accurate and current functional annotation of microarray probes is essential for the analysis and interpretation of the biological processes involved. As gene structures and functional annotation are updated in genome databases, the annotation attached to microarray probes must be updated so that scientists have access to the latest information with which to analyse their data.

**Results:**

We have designed a pipeline and database for the annotation of microarray probes using publically available databases. The pipeline is based on NCBI BLAST, Perl and MySQL. The pipeline was used to annotate a subset of 791 differentially expressed ArkGenomics chicken probes from an experiment involving chickens infected with the protozoan parasite *Eimeria*. Using our pipeline, 770 of the probes were assigned at least one entry in either the Ensembl, UniGene or the DFCI gene indices databases.

**Conclusion:**

The pipeline described here provides a simple and robust way of maintaining up-to-date and accurate annotation for microarray probes. The pipeline is designed in such a way as to be flexible and easy to update with new information.

## Background

The use of microarrays plays an important role in biomedical research, producing large quantities of data on genes that are differentially expressed under various conditions. Although annotation provided with the microarray may be current at the time of manufacture, regular reannotation of the microarray is essential to keep the annotation current. Additionally, probes may be designed from annotation based on incomplete genomes and incorrect or incomplete annotation. This may result in an incomplete coverage of the genome, non-specific probes, incorrect annotation, and orphan probes.

ProbeLynx [1] is a software system that has been published to accomplish the task of linking microarray sequences to annotation data. However, ProbeLynx uses certain tables directly from the Ensembl database and is therefore sensitive to schema changes. At the time of writing, ProbeLynx uses Ensembl version 47 (we are currently on version 52). Our objective is to design a flexible, up-to-date annotation pipeline that can be used to regularly update the annotation of microarray probes using publicly accessible databases which provide coverage of the genome. This paper is part of a workshop to compare different annotation pipelines, the results of which have been published in conjunction with this paper [2].

## Results

The pipeline has default filters such that only hits that match at greater than 80% identity across at least 20% of the length of the query sequence are counted. These values can be changed depending on requirements; for example, users would choose different values for a cDNA array compared to an oligo array. With these default values, 770 probes had at least one matching hit in at least one of the Ensembl, UniGene or DFCI gene indices databases.

Applying the selection criteria to the data presented here resulted in 750 probes having at least one matching hit in at least one of the Ensembl [3], UniGene [4] or DFCI gene indices [5] databases. The results from this study and the other studies on this dataset can be found on the EADGENE website [6].

### Ensembl

Using the Ensembl database, annotation could be provided for 472 probes (60%). Of those, 438 matched a single Ensembl gene id and 34 probes matched multiple genes. A total of 426 probes had perfect matches. Of these, 396 were unique hits. Gene descriptions were provided for 405 probes and 198 probes were matched to at least one Gene Ontology [7] term.

### DFCI gene indices

Using the DFCI gene indices, annotation was provided for 683 probes (86%). Of these, 249 matched a single gene index, 434 matched multiple indices, and 548 probes had perfect matches, 195 of which had single unique hits. Using the DFCI gene indices annotation, a gene description was provided for 466 probes and 66 probes were matched to at least one GO term [7].

### UNIGENE

Of the 791 probes, 715 (90%) could be assigned to at least one UniGene cluster, of which 593 were assigned uniquely (and therefore 122 were assigned to multiple clusters). Perfect matches were seen in 560 cases, of which 478 were unique. All 715 of the annotated probes had a cluster title (gene description).

## Discussion

When linking microarray probes to genome databases, we are attempting to do two things. Firstly, we are attempting to define just how many genes might be hybridising to each spot and contributing to the signal intensity. Secondly, we are attempting to inform scientists about gene function.

Ideally there should be a one-to-one relationship between probe and gene. However, this is clearly not the case. Using the selection criteria, the best results come from UniGene, where 75% of probes have a single, contributory gene; the worst results are from DFCI gene indices, where the figure is 31%. Probes with more than one hit may be due to shared domains, overlapping genes, misannotation, misassembly, low complexity regions, and/or repeat regions.

There are several reasons why probes may have no hits. The microarray used in this study was designed in 2005 using the first draft of the chicken genome, Ensembl version 30, and annotated with Ensembl version 42. Since then there have been 20 subsequent versions of the Ensembl database and a second draft of the chicken genome. Regular reannotation of the probes using the information provided with new genebuilds and Ensembl releases allows us to maintain up-to-date information. In addition, only the core Ensembl gene set was searched; had we searched against the genome itself, or against the EST gene set, the number of unannotated probes would be reduced. It is not surprising that the number of unannotated probes is lower in the two EST databases. However, even with UniGene, the best in terms of probe coverage, one in ten probes did not have a hit above the threshold. This may mean that the sequence that the probe was originally designed to is no longer publicly available (or never was) or that it did not meet the quality criteria applied before the database was built.

In terms of functional annotation, all three databases provided a functional description for over half of the probes. UniGene again performed the best, although no attempt has been made to judge the quality of the description. Disappointingly, a maximum of 25% of probes were assigned GO terms.

Future improvements in the assembly of the chicken genome and annotation should help to increase the level of annotation. The IMAD pipeline could be improved by allowing searches against the genome assembly, and against further databases such as the Ensembl EST genes, KEGG [8], and RefSeq [9]. This study is part of a workshop to compare different annotation strategies and the results of this have been published in conjunction with this study [2].

## Conclusion

We have created a pipeline that can be used to maintain the annotation of microarray probes using publicly available databases. The analysis of a set of differentially expressed probes revealed problems with annotation that may be due to a probe design based on incomplete annotation of the chicken genome. As improvements in the annotation of the chicken genome are made, improvements in the design of chicken microarrays are sure to follow.

## Materials and methods

### Software organisation

IMAD consists of a flexible relational database in MySQL, designed to store the hits of any set of sequences against any number of BLAST [10] databases, and any annotation associated with those databases; Perl scripts for downloading, updating and inserting Ensembl, UniGene and DFCI gene indices databases; a Perl API for querying the database programmatically; and a Perl CGI script for web-based querying.

### Workflow

The probe set was searched against multiple databases using NCBI BLAST, followed by parsing of the BLAST results. Where a single HSP exists between the query and hit, filters are applied and statistics are calculated and stored in the database. Where there are multiple HSPs, any overlap with respect to the query and the hit is removed. Statistics are then applied across all HSPs, filters applied and then stored in the database. Results (top hit for each probe for each database) in spreadsheet format are extracted using the API.

### Microarray dataset

The microarray used in this study was the Arkgenomics chicken 20 K oligo microarray, consisting 20,460 probes designed against a unique set of chicken transcripts in 2005, primarily 70 mer oligos [11]. A subset of 791 probes was selected for analysis in conjunction with the EADGENE post analysis workshop of microarray data [6] with the aim of evaluating several annotation pipelines for the quality of improved annotation. This represents a set of differentially expressed probes from an experiment of *Eimeria* infected chickens [12].

### Dataset sources for annotation

Ensembl chicken version 50, UniGene chicken build 39 and DFCI chicken gene indices version 11 were used. Gene Ontology terms were obtained through Ensembl BioMart [13]. These three databases provide

#### Selection criteria

Cutoff values for positive hits were any target with a contiguous matching stretch greater than 20 bases and an overall percentage identity greater than 80%. A perfect match is defined where there is a 100% match over the entire length of the oligo with the target sequence.

## Competing interests

The authors declare that they have no competing interests.

## Authors' contributions

MW and DP co-wrote IMAD.
